# Predictors and time to recovery from COVID-19 among patients attended at the treatment centers in Ekiti State, South West, Nigeria

**DOI:** 10.11604/pamj.2022.42.18.33791

**Published:** 2022-05-09

**Authors:** Oluwabunmi Samuel Ibitoye, Yusuff Akinkunmi Olasunkanmi, Tubosun Alex Olowolafe, Aderemi Temitayo Olabode, Mobolaji Modinat Salawu, Rotimi Felix Afolabi

**Affiliations:** 1 Ekiti State Hospitals Management Board, Ekiti, Nigeria,; 2 Epidemiology and Medical Statistics, Faculty of Public Health, College of Medicine, University of Ibadan, Ibadan, Nigeria,; 3 Health Promotion and Education, Faculty of Public Health, College of Medicine, University of Ibadan, Ibadan, Nigeria

**Keywords:** COVID-19, predictor, time to recovery, Nigeria

## Abstract

**Introduction:**

time to clinical recovery from COVID-19 infection and associated factors has not been explored in Nigeria. This study was conducted to assess the predictors and time to recovery from COVID-19 among patients attended to at the treatment centers in Ekiti State, South West.

**Methods:**

a facility-based retrospective cohort study was conducted between March 2020 to October 2021. Laboratory confirmed COVID-19 positive test result of 586 patients receiving treatment at the treatment centres in Ekiti were included. Data were extracted from COVID-19 intake forms and medical records of patients. Data were analysed using descriptive statistics and survival analysis methods including Cox proportional hazards regression model. Level of significance was set at 5%.

**Results:**

the mean age of the patients was 43.46 (SD 0.74) years. Forty-seven percent (47%) of the patients were aged 25-44 years, fifty-one percent (51%) were males. The median recovery time of COVID-19 patients was 21 days (IQR: 14-23). Being a male-patient (95% CI 20.46-21.54), older age (95% CI 20.14-21.86), not admitted in the hospital (95% CI 22.74-23.26), and associated multiple co-morbidities (95% CI 17.65-28.35) were associated with delayed recovery time. Predictors of recovery time of patients from COVID-19 infection were admission status (aHR: 0.71, 95%CI 0.56-0.88; p=0.002) and symptoms on admission (aHR: 0.81, 95%CI 0.66-0.99; p=0.020).

**Conclusion:**

patients with comorbidities, older and those not admitted were more likely to have a delayed clinical recovery from COVID-19. Knowledge of the predictors might help health professionals in risk stratification and better management of patients with COVID-19.

## Introduction

The World Health Organisation (WHO) declared COVID-19 outbreak as a public health emergency of international concern and a pandemic on 30^th^ January and 11^th^ March 2020 respectively [1]. Globally, more than 243 million people had been infected with COVID-19 and above five million deaths had been reported as of November 30, 2021 [2]. In Africa, the number of confirmed cases has continued to rise with over six million cumulative cases, representing 3% of global cases [3]. This has continued to stretch the health system as a result of pre-existing epidemics that are of critical concern [4].

In Nigeria, the index case of COVID-19 was reported on the 27^th^ February, 2020. Currently, COVID-19 infection has been reported in all the thirty-six states of the country including Federal Capital Territory. Of 3,298,966 samples tested, there were 211,921 confirmed cases, 203,248 survivors and 2,896 deaths as at November 1, 2021 [5]. The epicenter of the pandemic remains Lagos State with over 77,000 and least in Ekiti State with over 1,700 confirmed cases, both in the South West of the country [5].

COVID-19 continues to cause unprecedented morbidity and mortality, with a wide range of case-fatality rate across countries of the world including Nigeria. Clinical recovery from COVID-19 illness has been linked to demographics such as age, comorbidity, existing health care system and several unidentified factors [6,7]. The WHO report documented that the average recovery time from COVID-19 ranges from one to two weeks and three to six weeks for patients with mild and severe infections respectively [8]. A retrospective cohort study in Ethiopia among COVID-19 patients reported a median time to recovery of 18 days [9]. Another Ethiopian study among COVID-19 patients reported a range of 9 to 17 days with median recovery time of 13 days [6]. The average recovery time among COVID-19 patients in Indian states ranged from 5 to 36 days [10].

Nigeria Center for Disease Control (NCDC) reported that more than 95 percent of persons that tested positive for COVID-19 recovered from the disease [5]. However, the predictor and time to recovery from COVID-19 is yet to be documented in Nigeria. Knowledge of the predictors might help health professionals in risk stratification and better management of patients with COVID-19. This study was conducted to document the predictor(s) and time to recovery from COVID-19 among patients attended to at the treatment centers in Ekiti State, South West, Nigeria.

## Methods

**Study design and area:** a facility-based retrospective cohort study on recovery time from COVID-19 infection and its determinants among patients attended to from March 15, 2020 to October 21, 2021 to the treatment centres in Ekiti State, Nigeria. The secondary data utilised for this study was extracted from the registration logbook, COVID-19 intake forms and medical records of patients containing information on socio-demographic (such as age, sex among others), disease and past medical history, admission status clinical and laboratory characteristics of the patients. Ekiti state is located in the South-West region of Nigeria. As at 2006, the state had a total population of 2,398,957 [11]. In Ekiti state, the first case of COVID-19 was confirmed on the March 15, 2020. As at October 2021, Ekiti state had 1777 cases on record, with about 1726 patients who had recovered [5]. Ekiti State has three treatment centres for the management of COVID-19 patients. The treatment centres are located at Oba Adejugbe General Hospital Ado-Ekiti, Ekiti State University Teaching Hospital and Federal Teaching Hospital Ido-Ekiti.

**Study population, sample size and sampling technique:** this comprised of all patients who tested positive to COVID-19 using the antigen based rapid diagnostic test and reverse transcriptase polymerase chain reaction (RT-PCR), who were receiving treatment at any of the treatment centres in Ekiti State. A total of 597 patients case records were available within the study period, but only 586 had complete baseline information with outcome variable and were therefore included in the analysis. All COVID-19 patients attended to at the treatment centers from March 15, 2020 to October 21, 2021 and fulfilled inclusion criteria were included in this study. A total of 586 COVID-19 patients' case record date was analysed.

**Study variables:** the outcome variable for this study was time to recovery of patients from COVID-19 disease. The recovery time was estimated as number of days elapsed between confirmed date of positive test results and the subsequent confirmed date of negative test results among patients attended to at the COVID-19 treatment centres. In this study, patients who did not recover from the COVID-19 infection as at October 21, 2021 or those who died during the period of the study were right-censored. The explanatory variables considered for this study were patients´ socio-demographic and health characteristics which were: age (<24, 25-44, 45-64, ≥65 years), sex (male and female), admission status (admitted - for isolation and severity of illness; not admitted i.e. patients being managed from home as a result of mild symptoms, lack of space), symptoms on admission, oxygen therapy, presence of and the comorbidity conditions and recovery status (discharged after healthy certification, discharge against medical advice, death, referred to another centre). Recovery status was exclusively analysed based on patients that were discharged after health certification. Recall bias was envisaged in recollecting information regarding comorbid conditions. However, data relating to past medical history were extracted from available pre-existing patients´ records.

**Data analysis:** survival analysis techniques were used for this analysis. The “survival time” for patients who had recovered was the recovery time from confirmed cases of COVID-19 disease. Patients without confirmed negative test results at the end of the study were censored from the study. This was coded as zero (0); otherwise, it was coded one (1). The Kaplan-Meier survival method was used to describe the patient´s time to recovery from COVID-19 disease. The log-rank test was used to explore the association between COVID-19 recovery time and each of the independent variables. All significant variables (p<0.05) due to the log-rank test were thereafter included in the semi-parametric Cox proportional hazard regression to explore the predictors of COVID-19 recovery time. The adjusted hazard ratios (aHR) and their respective 95% confidence intervals are reported. All analyses were conducted at a 5% level of significance, using STATA 14.

**Ethical considerations:** ethical clearance was obtained from the Research and Ethics Committee for Planning and Statistics Department of Hospitals Management, Ekiti State (HMB/AD.567T/12). Permission was also obtained from the hospital administration for conduct of the research.

## Results

**Socio-demographic characteristics of patients:** the mean age of the patient was 43.46 (0.74) years. Of 586 participants, two hundred and seventy-nine of them (47.6%) were between 25-44 years while one hundred and fifty-nine (27.1%) were aged 45 to 64 years. Two hundred and ninety-nine (51%) of the patients were males. Four hundred and sixty-eight (79.9%) of the patients were admitted to the treatment centres, and three hundred and two of them (51.5%) had symptoms on admission. One hundred and fifty-two (25.9%) of the patients had a comorbidity at the point of admission. Sixty-six (11.2%) of the patients had hypertension, forty-seven had both hypertension and diabetes (8%) and about fifteen (2.6%) had only diabetes (Table 1).

**Table 1:** socio-demographic and clinical characteristics of COVID-19 cases attended at the treatment centres in Ekiti State

Variable	N (586)	%
**Age (years)**		
**Mean±SD**	43.49±0.74	
<24	63	10.8
25-44	279	47.6
45-64	159	27.1
≥65	85	14.5
**Sex**		
Male	299	51
Female	287	49
**Admission status**		
Admitted	468	79.9
Not admitted	118	20.1
**Symptoms on admission**		
Absent	284	48.5
Present	302	51.5
**Oxygen therapy**		
No	497	84.8
Yes	89	15.2
**Presence of comorbidity**		
No	429	73.2
Yes	157	26.8
**Comorbidity condition**		
No condition	434	74.1
Hypertension	66	11.3
Diabetes	15	2.6
Hypertension and diabetes	47	8
*Others	24	4.1
**Reason for being discharged**		
Discharged after healthy certification	539	92
+DAMA	10	1.7
Death	30	5.1
Referred to another centre	7	1.2

* i.e. Congestive Cardiac Failure (CCF), pneumonia, chronic ulcer, uterine fibroid, cancer, TB; +Discharge Against Medical Advise (DAMA)

**Treatment outcome among patients:** seven (1.2%) of the patients were still on treatment at the time of data collection, 30 (5.1%) of the patients died at the point of receiving treatment. At the end of the follow-up, 539 (92%) of the patients in the cohort recovered from the COVID-19 infection. Table 2 shows the chance of recovery of patients from the COVID-19 virus at different time points (days) after being attended to at the treatment centers. The probability of recovery from the COVID-19 virus in 10 days was 0.0814, with about 8% of the patients recovering from the virus in 10 days. Forty six percent of the patients recovered in 20 days, with most (90%) of the patients recovering from the virus at 25 days.

**Table 2:** probability of patients' clinical recovery from COVID-19 at different time point

Time (in days)	Probability of recovery	Standard error
5	0.0172	0.0054
10	0.0814	0.0114
15	0.3382	0.0199
20	0.4592	0.0211
25	0.9068	0.0127

**Figure 1:**
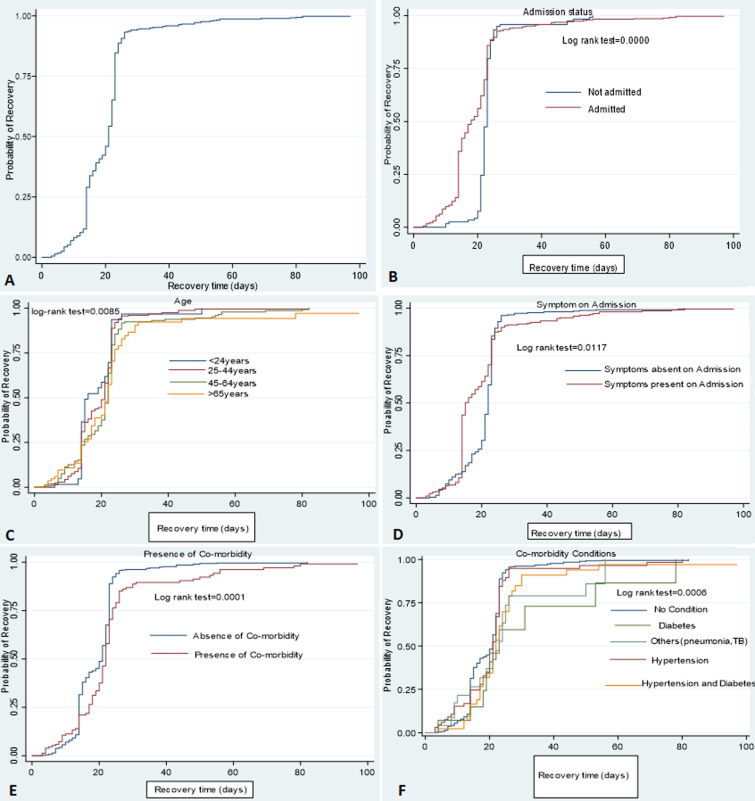
Kaplan-Meier recovery curve of COVID-19 patient with respect to the covariates

**Recovery rate and median recovery time from COVID-19:** of the 586 patients surveyed, 539 of them recovered within median recovery time of 21 (IQR: 14-23) days. The overall incidence rate of recovery was 4.70 per 100 (95% CI: 41.89-50.15) person-day observation. The overall median recovery time of COVID-19 patients was 21 days (IQR: 14-23) days presented in Figure 1A. Also, in Table 3, it can be observed that the median recovery time for male patients is 21 days (IQR: 15-23) as compared to the median recovery time for females which is 20 days (IQR: 14-23) at P<0.05. The median recovery time for patients aged < 24 year, 25-44 years, 45-64 years and ≥ 65 years was 16 days (IQR: 14-23), 21 days (IQR: 14-23), 22 days (IQR: 15-23) and 22 days (IQR: 14-24) respectively (P<0.05). The median recovery time for patients that were admitted was 18 days (IQR: 14-23) while for patients that were not admitted was 23 days (IQR: 22-23) at p<0.05. The median recovery time of patients without any form of co-morbidity condition was 21 days (IQR: 14-23) while the median recovery time of patients with co-morbidity condition was 22 days (IQR: 17-24) p<0.05. The median recovery time of patients with hypertension, diabetes and other forms of diseases (pneumonia, chronic ulcer, uterine fibroid, cancer, TB), diabetes and hypertension were 21 days (IQR: 17-23), 23 days (IQR: 19-53), and 22 days (IQR: 17-26) respectively p<0.05 (Table 3).

**Table 3:** median duration of recovery from COVID-19 with respect to the covariates for patients attended at the treatment centres in Ekiti State

Variables	Median (in days)	95%CI	Log-rank value	P-value
**Sex**				
**Overall**	21	20.46-21.54	4.03	0.0447*
Male	21	20.46-21.54		
Female	20	18.27-21.74		
**Age (years)**				
**Overall**	21	20.46-21.54	11.7	0.0085*
<24	16	12.76-19.24		
25-44	21	20.14-21.86		
45-64	22	21.41-22.59		
≥65	22	20.63-23.38		
**Admission status**				
**Overall**	21	20.46-21.54	17.71	0.000*
Admitted	18	16.39-19.61		
Not admitted	23	22.74-23.26		
**Symptoms on admission**				
**Overall**	21	20.46-21.54	6.36	0.0117*
Absent	22	21.63-22.37		
Present	15	13.26-16.74		
**Oxygen therapy**				
**Overall**	21	20.46-21.54	0.019	0.8897
No	21	20.58-21.42		
Yes	17	14.52-19.48		
**Presence of co-morbidity**				
**Overall**	21	20.46-21.54	15.79	0.0001*
No	21	19.91-22.10		
Yes	22	21.16-22.84		
**Co-morbidity condition**				
**Overall**	21	20.46-21.54	19.47	0.0006*
No condition	21	20.18-21.82		
Hypertension	21	20.11-21.89		
Diabetes	23	17.65-28.35		
Hypertension and diabetes	22	20.36-23.64		
Others i.e. CCF, pneumonia, chronic ulcer, uterine fibroid, cancer, TB	23	19.88-26.12		

* i.e. CCF, pneumonia, chronic ulcer, uterine fibroid, cancer, TB

The Kaplan-Meier survival curve representing the recovery time of COVID-19 with respect to patients´ health characteristics as covariates (admission status, symptoms on admission, presence of comorbidity, comorbidity condition) is constructed which are presented in Figure 1 (B-F). Kaplan-Meier survival curves were constructed to estimate the survival time based on different covariates to see the existence of differences in the recovery rate among categories of individual covariates. From the Kaplan Meier survival curve of individual covariates, there was no difference in the recovery rate of patients that received oxygen therapy and patients that didn´t receive oxygen therapy. However, there were different recovery rates among other covariates such as sex, age, admission status, symptoms on admission, presence of co-morbidity and co-morbidity condition (Figure 1 (B-F)). To show the significant difference in the covariates, a log-rank test was computed at a 5% significance level. There was a significant difference in survival status of patients to their sex, age, admission status, symptoms on admission, presence of co-morbidity and co-morbidity condition.

**Predictor of recovery time from COVID-19:** patients with symptoms on admission aHR = 0.81, 95% CI 0.66-0.99; p =0.02 were 0.81 times less likely to recover from the COVID-19 virus compared to patients without symptoms on admission. Patients that were not admitted aHR = 0.71, 95 % CI 0.57-0.89; p=0.002 were 0.71 times less likely to recover compared to those that were admitted. Patients with hypertension only aHR = 1.56, 95% CI 0.63-3.86; p=0.330 had 1.56 times higher chance of recovery time compared to patients with diabetes, hypertension with diabetes and other co-morbidity (pneumonia, chronic ulcer, tuberculosis) (Table 4).

**Table 4:** multivariable cox regression analysis of time to recovery and its predictors among patients attended at the treatment centres in Ekiti State

Variables	HR	95%CI	P-value	aHR	95%CI	P-value
**Sex**						
Male (ref)	1					
Female	1.16	0.98-1.36	0.077			
**Age (years)**						
<24 (ref)	1			1		
25-44	0.98	0.75-1.29	0.902	0.91	0.69-1.20	0.565
45-64	0.81	0.60-1.09	0.16	0.86	0.63-1.17	
≥65	0.89	0.64-1.23	0.472	0.87	0.58-1.32	
**Admission status**						
Admitted (ref)	1			1		
Not admitted	0.75	0.63-0.88	0.001*	0.71	0.56-0.88	0.002*
**Symptoms on admission**						
Absent (ref)	1			1		
Present	0.75	0.63-0.88	0.001*	0.81	0.66-0.99*	0.02*
**Oxygen therapy**						
No (ref)	1			1		
Yes	1.47	1.17-1.85	0.001*	0.82	0.60-1.12	0.2
**Presence of co-morbidity**						
No (ref)	1			1		
Yes	0.69	0.56-0.85	0.000*	1.83	0.72-4.60	
**Co-morbidity condition**						
No condition (ref)	1			1		
Hypertension	0.9	0.68-1.17	0.428	1.56	0.63-3.86	0.33
Diabetes	0.52	0.27-1.01	0.052	0.78	0.25-2.43	0.671
Hypertension and diabetes	0.62	0.44-0.87	0.006*	0.99	0.37-2.65	0.952
Others i.e. CCF, pneumonia, chronic ulcer, uterine fibroid, cancer, TB	0.58	0.36-0.92	0.021*	0.82	0.29-2.29	0.834

* statistically significant

## Discussion

The COVID-19 pandemic is a public health emergency with an unprecedented scale. With the changing dynamics of the epidemiology of COVID-19, an understanding of the survival time as well as associated factors is important. This study assessed the time to recovery from COVID-19 and its predictors among adults in Ekiti State, South western, Nigeria. The study found out that clinical recovery from COVID-19 infection was delayed in patients who were older, had comorbidities, and those not admitted in the hospital. In this study, more than two-fifth of the patients recovered from the virus at 15 days of receiving treatment. A study by [12], conducted in India reported similar findings of 29% of patients recovering at 15^th^ day of the treatment [13]. However, a study by Liu *et al*. in Australia reported that an estimate of 80% of the patients recovered after one month of treatment [14]. This difference in recovery rate maybe as a result of difference in weather condition, as reported by Mehmet in a research in Turkey that there is a negative correlation between temperature and transmission of COVID-19, which can also have an effect on patients´ recovery especially if the patient is asymptomatic [15].

Our study findings showed the median recovery time from COVID-19 virus was 21 days. This is similar to a study by Bi *et al*. and Zhon *et al*. in Shenzhen and Wuhan in China which reported a recovery time of 21 days from COVID-19 [16,17]. Wang *et al*. in another study in Wuhan reported an estimated median recovery time of 21 days which is similar to our study finding [18]. However, some other studies by Voinsky in Israel and Hu *et al*. in Shanghai documented lower median recovery time of 13.2 days and 11 days from COVID-19 respectively [19,20]. Furthermore, Abdella in a study carried out in Ethiopia reported an estimated median recovery time of 19 days from COVID-19 [21]. This time to viral load clearance is also similar to our study findings. The disparity in median recovery time in these studies could be due to study site, study sample size, changes in the country's socio-economic situation as a result of the pandemic, disease severity, type of specimen tested, care and treatment given.

In this study, we found that female patients will most likely recover before the male patients because the predictive recovery time was lesser for female patients. This finding was similar to other studies conducted in Israeli, Wuhan and Ethiopia [16-21]. However in another study by Liu *et al*. in Australia, there was no significant association between sex of the study participant and time to recovery [14]. This can be explained in terms of sex-related differences that exist between males and females; as the latter are believed to have stronger immune system compared to males, and higher level of cortisol and oestrogen which are beneficial [22,23].

Our study further revealed that the presence of comorbidity is an independent risk factor that can delay the recovery of patients from COVID-19. This finding was supported by different studies which identified the presence of comorbidity in COVID-19 patients led to delay in recovery [20,24,25]. Although, there were other studies that reported no significant association between the presence of comorbidity and time to recovery from COVID-19 [26,27]. The observed difference could be as a result of varying treatment guidelines for the management of these comorbidities in the different study site. In addition, there is the possibility of variation in patients´ response to treatment which may have an effect on recovery time form COVID-19.

In this study, we found a significant association between age and time to recover from COVID-19, which showed that the median recovery time for patients below the age 24 years was 16 days while older patients aged 45 years and above had median recovery time of 22 days. A few other studies conducted in China reported similar findings [20,26]. However, Abdella in a study on time to recover and its predictors among adults hospitalized in Ethiopia found no association between age and viral clearance [21]. In addition, Qi *et al*. in a study on factors associated with the duration of viral shedding in adults with COVID-19 outside of Wuhan, Chins reported no significant difference between age duration of viral shedding [28]. Natural ageing process is characterized by a decline in physiological functions and weakened immune system. It is sometimes associated with comorbid disorders which also predispose the aged to elongate recovery time and hence could result in poor clinical outcomes in older adults with COVID-19 [29].

The predictors time to clinical recovery of patients with COVID-19 infection within the median time of recovery were admission status, symptoms on admission and presence of comorbidity. COVID-19 patients that were not admitted were less likely to recover early as compared to those that were admitted. Patients with symptoms on admission were also less likely to recover quickly from the COVID-19 compared to patients without symptoms on admission. Patients with hypertension only has little higher chance of recovery within the median time of recovery compared to patients with diabetes, hypertension with diabetes and other co-morbidity such as pneumonia, chronic ulcer, tuberculosis. Research has documented that COVID-19 complications is severe among people with underlying health conditions and treatment outcomes are always unfavourable [30-32]. Leulseged *et al*. reported that presence or absence of co-morbid symptoms among COVID-19 patients was a significant factor that influenced recovery days [33]. This is also similar to a study by Tolossa which reported that symptoms and comorbidity were key factors that determine COVID-19 treatment recovery time [34]. Osibogun in another study in Lagos, Nigeria found out that comorbidity and the level of its severity were the cause of patients´ deaths and recovery [35].

This is the first study to document predictors and time to recover from COVID-19 infection in Nigeria. However, some limitations are noted. Firstly, this was a retrospective study which limited further exploratory analysis because some other useful information as regards patients´ socio-demographic information, clinical features/state and other laboratory findings might not have been captured. Secondly, recall bias was envisaged in this study as patients may find it difficult to recall information in the past as regards their co-morbid conditions that could be related to COVID-19 viral clearance. However, we were able to assess some data relating to past medical history from available pre-existing patients´ records.

## Conclusion

This retrospective cohort study showed that the recovery time from COVID-19 infection is long and is particularly longer in patients with other co-morbidities, older adults and patients that were not on admission. Therefore, aged COVID-19 patients, and persons with one or more co-morbidities should be given priority for COVID-19 preventive interventions. In addition, these findings will assist the clinician to triage and administer appropriate and timely treatment to COVID-19 patients which gives a favourable clinical outcome.

### What is known about this topic

- Epidemiology of COVID-19 infection has been exolained in many published literatures;
- Prevalence and clinical symptoms associated with COVID-19 infection has been established.

### What this study adds

- This study has added scientific evidence on time to clinical recovery of patients from COVID-19 infection. It has also demonstrated significant predictors of patients' clinical recovery from COVID-19 infection.
